# Implementation of the European Society of Cardiology 0/3-hour accelerated diagnostic protocol, using high sensitive troponin T: a clinical practice evaluation of safety and effectiveness involving 3003 patients with suspected acute coronary syndrome

**DOI:** 10.1136/openhrt-2023-002366

**Published:** 2023-12-26

**Authors:** James Daniel Hatherley, Thomas Salmon, Paul O Collinson, Aleem Khand

**Affiliations:** 1 Liverpool Centre for Cardiovascular Sciences, University of Liverpool, Liverpool, UK; 2 Department of Cardiology, Royal Liverpool and Broadgreen Hospitals NHS Trust, Liverpool, UK; 3 Department of Cardiology, Aintree University Hospitals NHS Foundation Trust, Liverpool, UK; 4 Clinical Blood Sciences, St George's University Hospitals NHS Foundation Trust, London, UK; 5 Department of Cardiology, Liverpool Heart and Chest NHS Foundation Trust, Liverpool, UK

**Keywords:** coronary artery disease, acute coronary syndrome, chest pain, myocardial infarction, delivery of health care

## Abstract

**Background:**

There have been relatively few studies detailing the real-world effectiveness and safety of accelerated diagnostic protocols (ADP), using high sensitivity cardiac troponin (hs-cTn).

**Objective:**

To analyse the safety and effectiveness of early emergency department (ED) discharge following implementation of the European Society of Cardiology (ESC) 0/3-hour ADP for suspected acute coronary syndromes (ACS).

**Method:**

We prospectively studied 2 cohorts of consecutive suspected ACS presentations to ED before (n=1642) and after (n=1376, 2 centres) implementation of the ESC 0/3-hour ADP incorporating limit of detection rule out. Safety was defined by MACE (major adverse cardiac events) inclusive of type 1 myocardial infarction (MI) in patients discharged from ED, and clinical effectiveness by percentage ED discharge. Continuous variables and categorical data were evaluated by independent t-test and χ^2^ test, respectively. Time-to-event data were analysed as survival data and converted to Kaplan-Meier curves for interpretation.

**Results:**

In the preimplementation period, there was a higher prevalence of MI. Discharge from ED increased by >100% (from 27.1% to 56.5% of the cohort) with no safety signal (MACE rate 4/444 (0.9%) vs 4/769 (0.52%), p=0.430 for the 2011 and 2018 cohort, respectively). This correlated with a marked reduction in length of stay overall but a more modest reduction for those discharged from ED (6 hours 10 min vs 5 hours 25 min, p<0.001) for the 2011 and 2018 cohort, respectively. There were improvements in presentation to blood draw (163–90 min, p<0.001). Time from presentation to first ECG actually increased (16.2 vs 31.2 min, p<0.001). Analysis of hs-cTn values and ECGs revealed a maximum ED discharge rate of 69%, by applying the 0/3-hour protocol, implying potential for increasing safe ED discharge.

**Conclusions:**

Implementation of an ADP with hs-cTn is safe and effective for early rule-out and discharge of suspected ACS but require considerable resources and education to optimise maximal patient flow.

**WHAT IS ALREADY KNOWN ON THIS TOPIC**

- High sensitivity cardiac troponin assays have led to the development of multiple accelerated diagnostic pathways (ADPs), including the European Society of Cardiology’s (ESC) 0/3-hour protocol, for the rule out of myocardial infarction. The safety of these pathways is well established but there are less data looking into the effect on patient flow in busy emergency departments (EDs).

**WHAT THIS STUDY ADDS**

- Patients on a local ADP had significantly reduced length of stay when compared with the pre-ADP group, with no compromise on patient safety. Implementation of the ADP into a busy ED made further logistical improvements, such as reduction in time to first blood draw. There was, however, a lengthened time to first ECG.
- The study acknowledges that the troponin result is only part of the patient’s journey and focussing on one aspect may lead to delays in other critical areas. As further reductions in the troponin sampling interval are advocated, ED clinicians must reflect on the patient journey in their department to ensure that these improvements impact on patient care.

**HOW THIS STUDY MIGHT AFFECT RESEARCH, PRACTICE OR POLICY**

- Clinicians can be confident in the safety of the ESC 0/3-hour ADP. Adoption of this pathway is well established and has clear benefits in reducing length of stay.
- This study reinforces the need for robust, real world, randomised data comparing rapid diagnostic algorithms. It not only assesses their safety but ensures the reduction in troponin sampling time is reflected in the length of stay in ED. The shorter the sampling interval, the more likely competing factors other than troponin will delay decision-making and discharge. The extent of this is currently unknown.

## Introduction

Chest pain suggestive of acute coronary syndromes (ACS) is common presentations to the emergency department (ED), accounting for up to 10% of all ED consultations.^1–3^ Although chest pain is common, less than 10% of patients are diagnosed with myocardial infarction (MI).^4–6^ Excluding MI or other serious pathology early has clear advantages for both the patient and ED. The patient is reassured earlier, reducing exposure to unnecessary interventions and time in ED. The institution gains by improving patient flow and thereby reducing ED overcrowding, a known cause of increased morbidity and mortality especially in suspected ACS. For those with MI, earlier diagnosis leads to earlier monitoring, treatment and risk stratification.^7 8^

**Figure 1:**
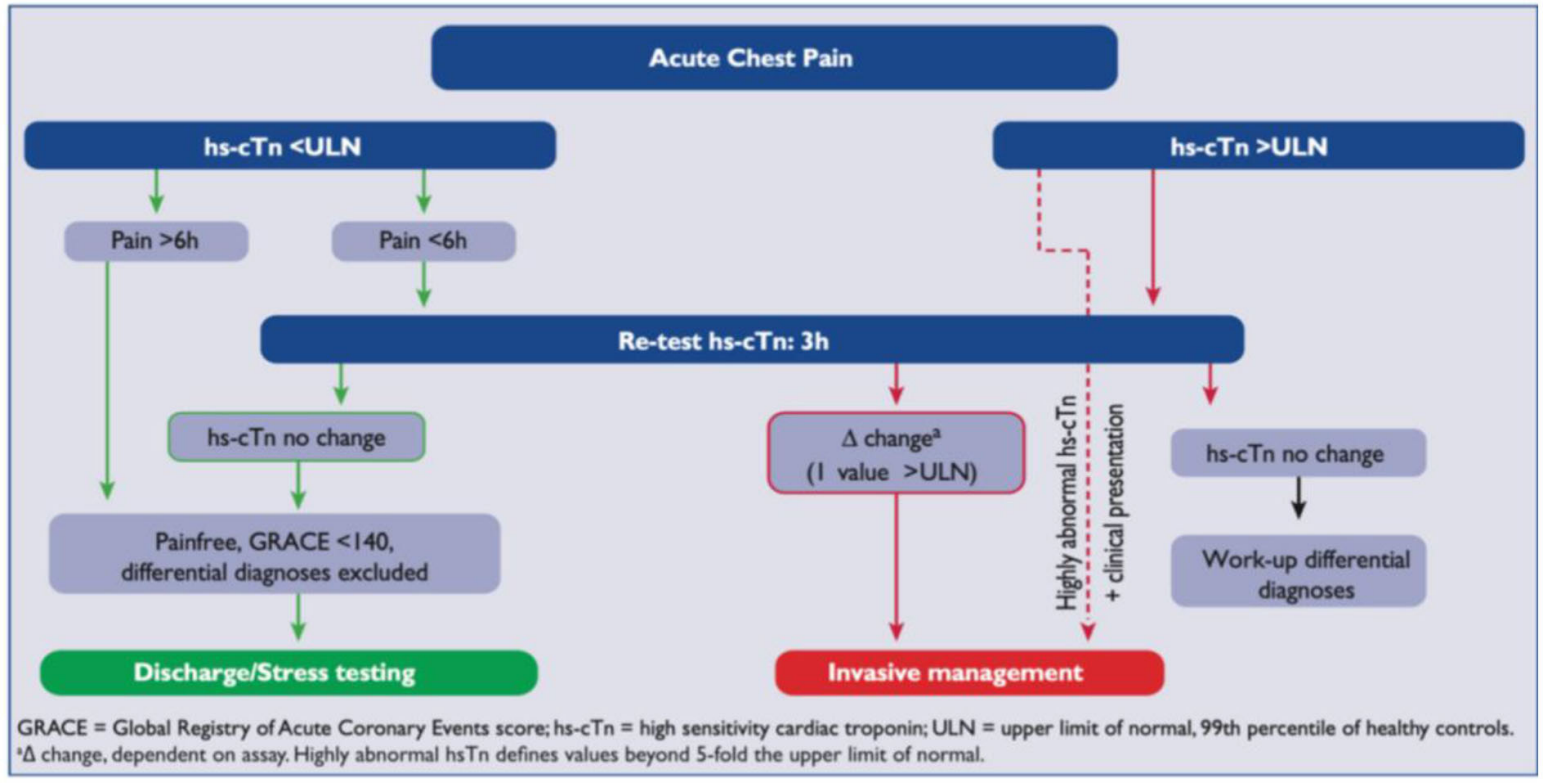
ESC 0/3-hour protocol for suspected acute coronary syndrome. ESC, European Society of Cardiology.

The measurement of cardiac troponin (cTn) is central to the diagnosis and universal definition of MI.^8 9^ The development of high sensitivity cTn (hs-cTn) has led to improved diagnostic sensitivity and earlier exclusion of MI via rule-out algorithms.^10^ Improved sensitivity has, however, come at the cost of reduced specificity with detection of myocardial injury in a range of other clinical conditions than MI. Due to the improved analytical performance of hs-cTn assays, concentrations can now be measured in most healthy individuals. To be designated a high sensitivity assay, the 10% coefficient of variation must be below the 99th percentile with the ability to detect at least 50% of a healthy population.^11^ The Roche highly-sensitive Troponin-T (hs-TnT) assay has been shown to fulfil these criteria.^6 12^ The development of hs-cTn assays has enabled the European Society of Cardiology (ESC) to recommend an accelerated diagnostic protocol (ADP) for patients with suspected non-ST elevation ACS.^7^ This rule-out algorithm uses hs-cTn samples taken at 0 and 3 hours (figure 1). This pathway was based on multiple prospective studies.^12–14^

Maximising the potential of hs-cTns has been slow with patchy uptake of ADPs.^15^ Consequently, there is wide variation in discharge rates for suspected ACS. Reducing this variation in discharge was one of the key National Health Service (NHS) priorities manifested by advanced access collaboratives.^16^ We sought to study the impact and challenges of the ADP when implemented in clinical practice. We benchmarked metrics against a previous cohort of patients, who despite the use of hs-cTns still underwent 6-hour and 12-hour repeat troponin testing. We wished to understand the impact of implementing an ADP, particularly focusing on patient flow and discharge rates. We studied the patient journey in suspected ACS, preimplementation and postimplementation of the ADP.

**Figure 2:**
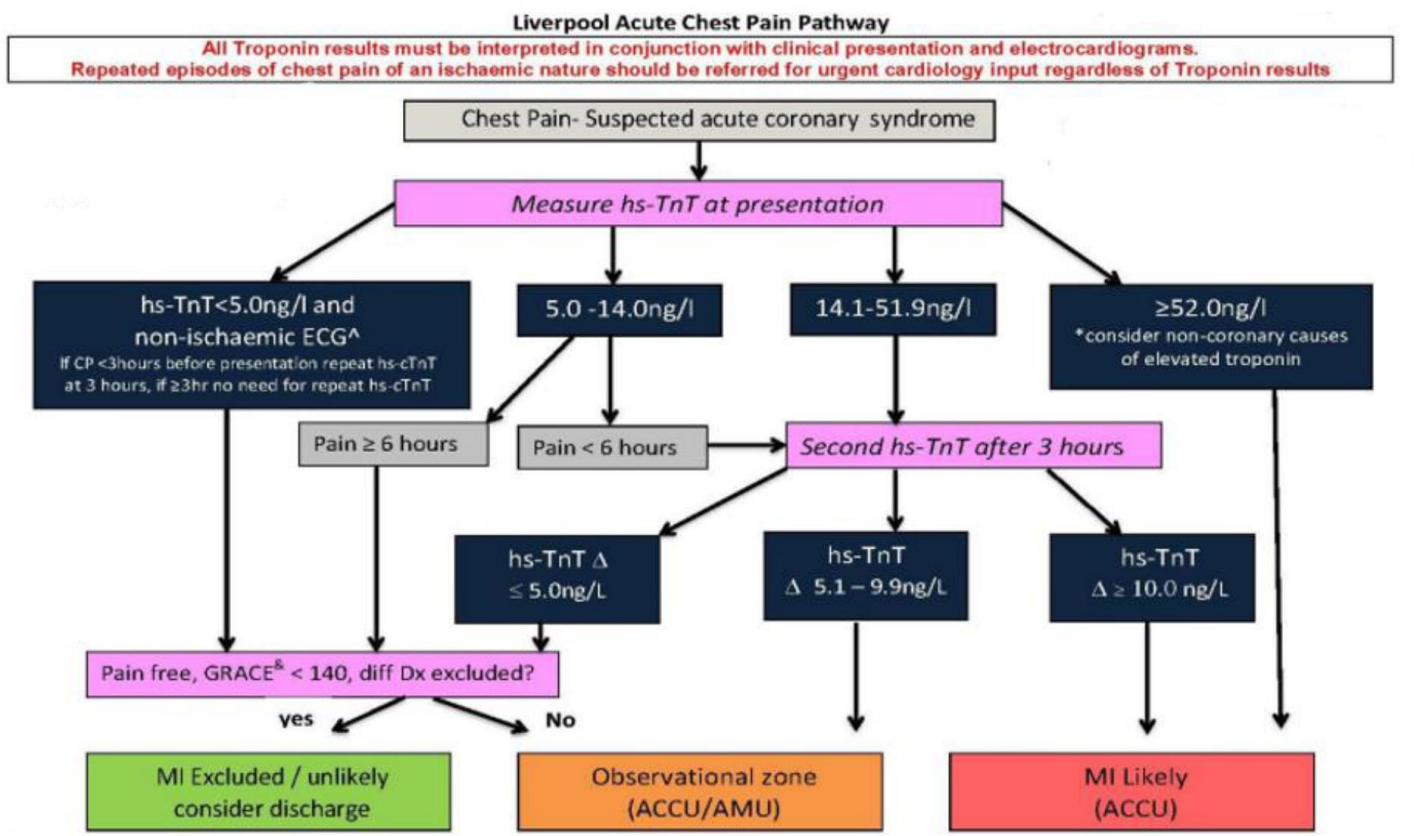
Liverpool 0–3 hours pathway for suspected acute coronary syndrome. ^&^GRACE score predicts six month mortality following acute coronary syndrome. ˆThis cohort of patients are potentially suitable for single sample rule out if CP onset over three hours from blood sampling. ACCU, Acute Cardiac Care Unit; AMU, Acute Medical Unit; CP, chest pain; GRACE, Global Registry of Acute Coronary Events; hs-TnT, high sensitivity troponin T; MI, myocardial infarction.

The Liverpool Acute Chest Pain Pathway (LACPP) (figure 2) is an adaptation of the ESC 3-hour rule-out pathway. This approach is based on evidence from multiple perspective studies and a meta-analyses.^3–6 17 18^ Prior to the introduction of the LACPP, patients had troponin sampled at 6 hours, and repeated at 12 hours if greater than the 99th percentile. If blood was sampled before 6 hours of the chest pain onset, the samples were repeated at 6 hours, regardless of the initial result. Rather than an agreed proportional increase or decrease in troponin level over 6 hours, clinical judgement was used to rule-in MI. There was no single sample rule-out based on limit of detection (LOD).

### Aim

To assess the safety of the LACPP when implemented in clinical practice.

Furthermore, to evaluate the impact on discharge from ED and the length of stay (LOS) in ED. Safety is defined using the composite primary endpoint of major adverse cardiac events (MACE), in those discharged from the ED. A direct comparison was made with the preimplementation phase.

## Method

We undertook a two-centre quality improvement project (QIP) with audit approximately 1 year after implementation of the new ADP in 2018. All consecutive patients presenting with suspected ACS were prospectively enrolled in the study. Suspected ACS was defined as symptoms of chest pain, with a sample taken for troponin measurement and an ECG performed at presentation. This 2018 cohort was compared with a historical prospective cohort from 2011 enrolled using the same criteria. The principle aim of the historical cohort was to evaluate and compare discharge strategies before and after introduction of the ADP using hs-cTnT.^6^

We established in the 2011 cohort, with patients tracked nationally, that no subsequent adjudicated MI, to 1 year, presented outside the local region. We tracked the 2018 validation cohort regionally, rather than nationally.

For both cohorts, we collected data on time of troponin sampling relative to time of presentation, the results of the ECG and the troponin results and kinetics. In addition, length of hospital stay was documented.

### Efficiency of process

We collected specifically time to first ECG after presentation, time to first blood sample and second sample, discharge time relative to presentation as metrics of efficiency of the process.

The 2011 preimplementation cohort consisted of 1637 consecutive patients in a single trust (Aintree University Hospital (AUH)). The data in this group were collected retrospectively. The 2018 postimplementation cohort was 1366 consecutive patients across 2 hospital trusts (AUH and the Royal Liverpool University Hospital). Prospective data collection started 1 year after implementation of the ADP. This allowed time for staff education and training across both sites.

### Adjudication of MI

All patients with elevated hs-TnT at the index event, or any readmission up to 1 year in any hospital in England (2011) or the NW England (2018) underwent two physician adjudication for type 1 or type 2 MI using all available data. To ensure consistency, patients for both cohorts were adjudicated according to the third universal definition of MI, using hs-TnT as a biomarker of myocardial necrosis.^19^ This included results of investigations (eg, coronary angiography, echocardiography) up to 6 weeks following presentation. A third cardiologist acted as a tie-breaker in case of disagreement over diagnosis.

### Follow-up

NHS digital was employed using the UK unique linked hospital database to track any patients for subsequent admission using a range of International Classification of Disease 10th Revision (ICD-10) codes for cardiac or cardiac related conditions including chest pain, MI, angina. Online supplemental table S1 details codes used to input subsequent presentations, admissions, coronary revascularisation procedures to any English hospital.

10.1136/openhrt-2023-002366.supp1 Supplementary data

### Outcome

The primary outcome data were MACE and/or type 1 MI at 6 weeks. MACE is a composite endpoint comprising of MI, urgent revascularisation (via percutaneous coronary intervention or coronary artery bypass grafting) or all cause death. Readmissions for unstable angina were not included in the composite endpoint. As a secondary endpoint, we looked at discharge rates and time to discharge from ED. We also looked at time from presentation to first ECG and first hs-TnT blood draw as performance metrics.

### Statistics

Continuous variables are presented as medians (IQR) and categorical variables are presented as n (%). Continuous variables were evaluated using independent t-test and Categorical data by χ^2^ test. All time-to-event data were analysed as survival data. These data were converted into Kaplan-Meier curves for interpretation. Log rank analysis was undertaken between the two curves to demonstrate statistical significance.

## Results

### Demographics

Table 1 details the demographics and epidemiology of the two cohorts. The 2018 cohort was younger and had less cardiovascular risk factors. There was a higher proportion of patients with a history of MI in the 2011 cohort (19.6% vs 13.6% p=0.001).

**Table 1:** Demographics and efficiency

Cohort	2011 (n=1637)	2018 (n=1366)	P value
Demographics			
Male, n (%)	858/1637 (52.4%)	708/1366 (51.8%)	0.750
Age (median, IQR, full range)	(n=1640) 58, (46–72), (18–102)	(n=1376) 55, (42–70), (16–101)	<0.001
≥3 risk factors, n (%)	572/1637 (34.9%)	393/1257 (31.3%)	0.038
With previous MI, n (%)	321/1637 (19.6%)	186/1365 (13.6%)	0.001
With previous PCI or CABG, n (%)	91/1637 (5.6%)	162/1361 (11.9%)	<0.001
With previous CVA, n (%)	118/1637 (7.2%)	67/1356 (4.9%)	<0.001
Timings			
CP to presentation	(n=1621) 9 hours 57 min, (IQR: 2 hours 35 min to 48 hours)	(n=1339) 8 hours 50 min, (IQR: 2 hours 56 min to 24 hours 37 min)	<0.001
Presentation to first ECG (min)	(n=1585) 16.2, (IQR: 7.2–33.0)	(n=1287) 31.2, (IQR:16.2–60.0)	<0.001
Presentation to first hs-TnT blood draw (min)	(n=1636) 163, (IQR: 89–291)	(n=1364) 90, (IQR: 52–142)	<0.001
At least two hs-TnT, n (%)	676/1637 (41.3%)	529/1366 (38.7%)	0.152
Time between first and second hs-TnT	(n=676) 7 hour 19 min, (IQR: 5 hours 46 min to 11 hours 39 min)	(n=529) 3 hours 54 min, (IQR: 3 hours 10 min to 5 hours 24 min)	<0.001
LOS	(n=1637) 16 hour 19 min (IQR: 6 hours 30 min to 66 hour 51 min)	(n=1354) 7 hours 7 min (IQR: 4 hours 13 min to 25 hours 33 min)	0.046
Time in hospital if discharged without admission	(n=484) 6 hours 10 min (IQR: 4 hours 31 min to 8 hours 15 min)	(n=768) 5 hours 25 min (IQR: 3 hours 42 min to 8 hours 15 min)	<0.001

CABG, coronary artery bypass graft; CP, chest pain; CVA, cerebrovascular accident; ECG, electrocardiogram; hs-TnT, high-sensitivity troponin T; IQR, interquartile range; LOS, length of stay; MI, myocardial infarction; PCI, percutaneous coronary intervention.

### Measures of efficiency

**Figure 3:**
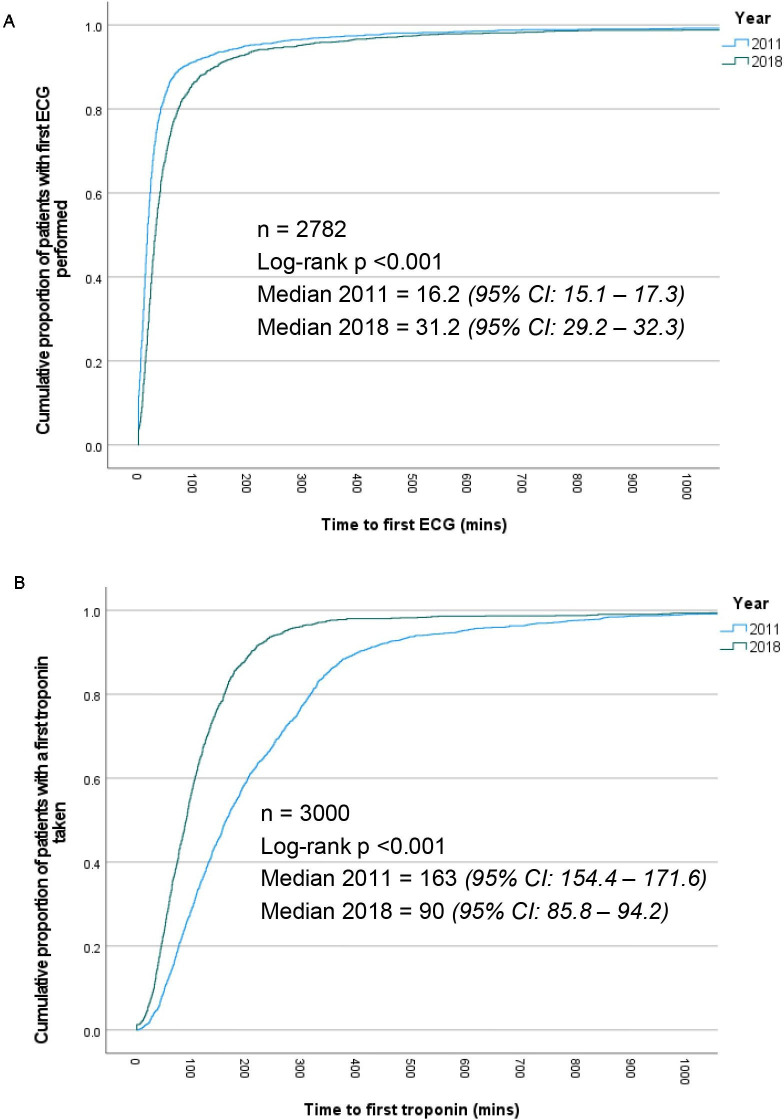
(A) Kaplan-Meir curve comparing time from admission to ECG between the two cohorts. (B) Kaplan-Meir curve comparing the time from admission to first troponin being taken in minutes between the two cohorts.

Time from presentation to first ECG was longer with the LACPP (figure 3A). However, time from presentation to first hs-TnT draw was significantly shorter following the introduction of the LACPP (figure 3B). Time between first and second hs-TnT draw was significantly shorter after the introduction of the LACPP (7 hours 19 min, (IQR 5 hours 46 min to 11 hours 39 min) vs 3 hours 54 min, (IQR 3 hours 10 min to 5 hours 24 min) p<0.001).

**Figure 4:**
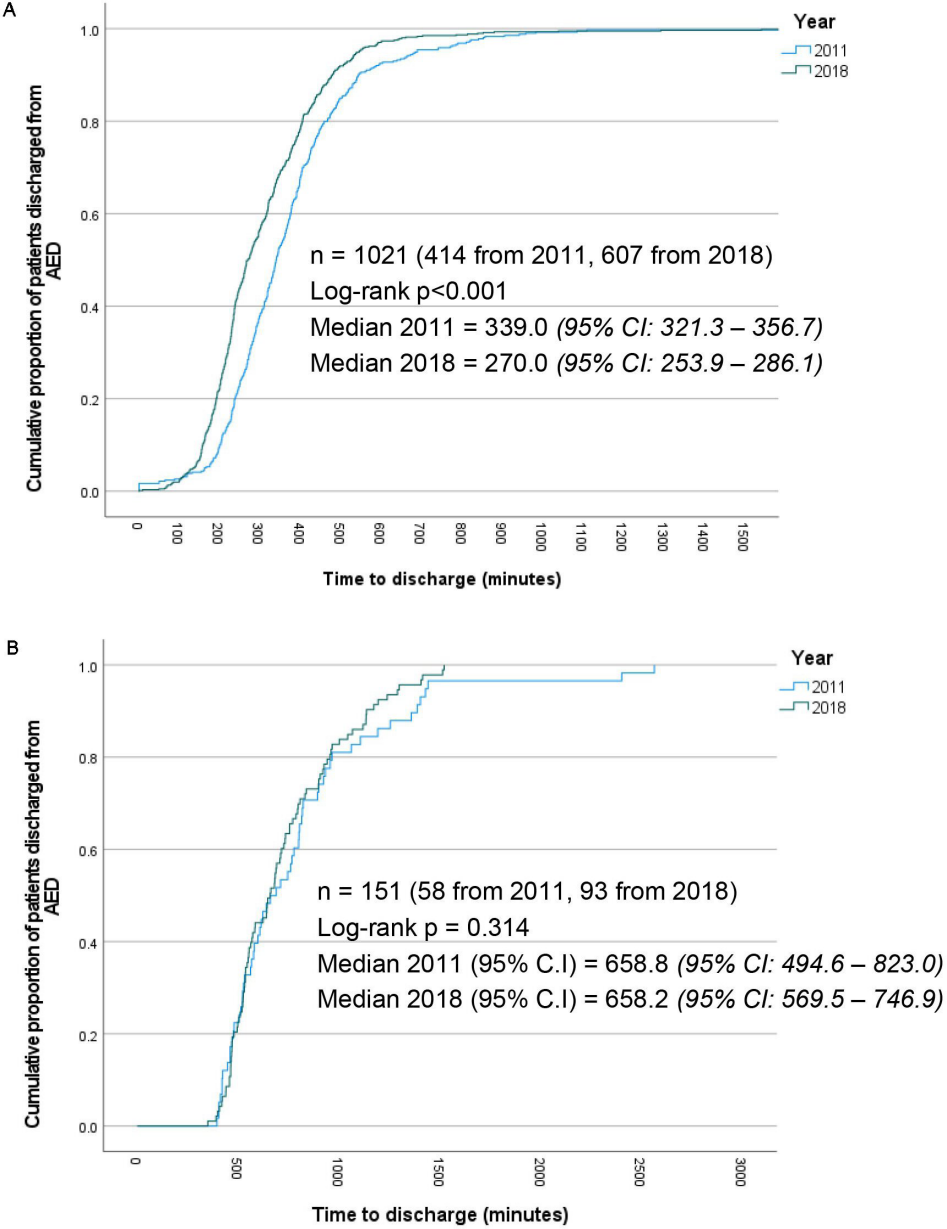
(A) Kaplan-Meir curve showing time from presentation to discharge, in patients discharged directly from the AED within 6 hours of their last troponin (minutes). (B) Kaplan-Meir curve showing time to discharge from AED for all patients not admitted to hospital, discharged between 6 and 24 hours after their last troponin (minutes). AED, Accident and Emergency Department.

The LACPP significantly reduced LOS in hospital if discharged from the ED, when limiting patients included to those who were discharged within 6 hours of their last troponin being taken (figure 4A). If a patient was not discharged within 6 hours of the troponin, then it was deemed unlikely the result alone hindered their discharge. Figure 4B indicates unsurprisingly discharged times were not improved in this cohort. The strong suspicion is that the troponin result was not hindering their discharge, but a range of other factors.

### Safety of accelerated diagnostic protocol

Table 2 outlines safety and outcome data. The 2011 preimplementation cohort was a sicker population in terms of cardiovascular disease with a higher index MI rate. Overall MACE at 30 days was significantly greater in 2011 (preimplementation) to 2018 postimplementation (13.9% vs 7.1%: p<0.001). Patients in the 2018 cohort had a significantly higher rate of discharge from ED (27% vs 57%: p<0.001). For those discharged direct from ED, MACE at 30 days was similar between cohorts (0.9% vs 0.5%: p=0.430). All-cause mortality at 30 days was higher in the 2011 cohort but not significant (1.5% vs 0.7%: p=0.064).

**Table 2:** Outcome and safety

Cohort	2011 (n=1637)	2018 (n=1366)	P value
Final diagnosis and outcome			
Index diagnosis of T1MI, n (%)	172/1637 (10.5%)	61/1366 (4.5%)	<0.001
Index diagnosis of T1 or T2MI, n (%)	192/1637 (11.7%)	82/1366 (6.0%)	<0.001
Index diagnosis of UA, n (%)	500/1637 (30.5%)	236/1366 (17.3%)	<0.001
Admission to hospital, n (%)	1185/1637 (72.4%)	592/1361 (43.5%)	<0.001
Discharged from ED, n (%)	444/1637 (27.1%)	769/1361 (56.5%)	<0.001
Safety			
Death 30 days, n (%)	22/1637 (1.5%)	9/1366 (0.7%)	0.064
MACE at 30 days, n (%)	219/1637 (13.9%)	97/1366 (7.1%)	<0.001
MACE at 30 days for those discharged without admission to hospital, n (%)	4/444 (0.9%)	4/769 (0.5%)	0.430
T1 or T2MI at 30 days for those discharged without admission to hospital, n (%)	3/444 (0.7%)	3/769 (0.4%)	0.495
Adjudicated T1 or T2MI on index admission for those discharged without admission to hospital, n(%)	3/444 (0.7%)	2/769 (0.3%)	0.276
No of patients discharged without admission to hospital, who were adjudicated T1 or T2MI on index admission, n (%)	3/192 (1.6%)	2/82 (2.4%)	0.620

MI, revascularisation or all cause death. P values calculated using χ2 test of independence.

ED, emergency department; MACE, major adverse cardiac events; NA, not available; T1MI, type 1 myocardial infarction; UA, unstable angina.

## Discussion

The high proportion of patients with chest pain in ED means that prompt assessment is essential to flow within the department. The LACPP has improved LOS in the ED without compromising patient safety.

The LACPP was adapted from the ESC 3-hour rule-out pathway. Patients in the 2011 cohort were a higher-risk population than the 2018 cohort. Unsurprisingly, there were more type 1 MIs at index presentation, higher rates of MACE and all-cause mortality at 30 days. Despite this, in patients who were discharged from ED, rates of MACE, type 1 or type 2 MI at 30 days were similar between groups. This suggests non-inferiority with regard to safety of rule out between the two cohorts. In a similar study Sandeman *et al*^4^ analysed 10 315 consecutive patients presenting with symptoms suggestive of ACS. Patients who presented prior to June 2016 were managed using the standard protocol. This involved hs-TnT sampling on presentation, with a repeat at 6 hours and potentially a further repeat at 12 hours. The earliest point of rule-out was 6 hours. Patients presenting after June 2016 were managed using a rapid 3 hour rule-out pathway. In these two cohorts they found that there was no difference in all-cause mortality or cardiovascular mortality at 30 days or 1 year.

Use of the LACPP significantly reduced patients’ total LOS. The most obvious explanation is that blood sampling is done at a shorter interval. It also has the capacity to rule out MI with one hs-TnT measurement. It is widely appreciated that LOS is also influenced by how busy ED departments can be, impacting on time to assessment, treatment and discharge. Despite this, improvements were still seen with the LACPP.

Similar results were found by Sandeman *et al*.^4^ They found a significantly lower LOS for patients on a 3-hour rule-out pathway as opposed to the previous 6-hour pathway. In a stepped-wedge cluster RCT in Scotland,^20^ 31 492 consecutive patients were analysed; 14 700 in the standard care arm and 16 792 in the intervention arm. The standard care involved hs-TnI (Abbot Architect troponin I) sampling at presentation and then repeated at 6–12 hours if required. The intervention arm had a single sample rule out if initial hs-TnI was <5 ng/L and symptom onset was >2 hours prior. Samples were repeated at 3 hours with the potential to rule out MI if the delta values were met. In this trial, the intervention arm reduced LOS from 10.1±4.1 hours to 6.8±3.9 hours (p<0.001). Discharge rates from the ED were increased from 50% to 71% (OR 1.59 (95% CI 1.45 to 1.75%). There was no significant difference in MI or cardiac mortality between the groups at 30 days and 1 year.

The LACPP was introduced following endorsement in the 2015 ESC guidelines for management of patients without persistent ST-elevation on ECG.^7^ This project provides an insight into the practice of the hospital trusts it was undertaken in. However, it must be recognised that these recommendations are somewhat historic. The two most recent guidelines in 2020 and 2023 have endorsed and now recommend 0/1-hour and 0/2-hour rule out algorithms.^21 22^ At the time of data collection, these more rapid rule out algorithms were in their infancy.

The ability for the LACPP to reduce LOS and burden on EDs is dependent on reduced intervals to repeat sampling. With the newly endorsed 0/1-and 0/2-hour algorithms, this interval is further reduced. This is an exciting prospect but not exempt from wider ED pressures. It relies on timely sampling and prompt action once the result is available. There are various points in the process where there could be delays.

An observational implementation study by Twerenbold *et al*^23^ assessed the effect of the 0/1-hour rule out algorithm on ED LOS. In 2296 patients with suspected ACS, the median LOS in the ED from admission to either transfer or discharge was 150 min. There was no comparator group in this study but given that average time to discharge was less than 3 hours, it is likely that this would be superior to a 0/3-hour algorithm. This study, however, was conducted in private healthcare systems so these results are not directly applicable to public healthcare systems like in the UK. Further research is required to assess feasibility of the 0/1-hour algorithm in this setting.

A randomised controlled trial of 3378 patients compared the 0/1-hour rule out algorithm with a masked, non-high sensitive 0/3-hour algorithm.^24^ Patients managed with the 0/1-hour and the 0/3-hour algorithm had a median ED LOS of 4.6 hours (IQR 3.4–6.4) and 5.6 hours (IQR 4.0–7.1) respectively (p=0.001). Interestingly, the median LOS of patients managed in this trial’s 0/3-hour arm was very similar to those in our QIP, 5.6 hours and 5.5 hours, respectively. This indicates that a similar improvement may be possible with a 0/1- hour algorithm.

Time to blood sampling improved following LACPP implementation, the median time was still 90 min. This could be improved with staff training in triage of suspected ACS patients, including timely troponin sampling at the front door. In addition, phlebotomists present in ED may ease the pressure on other clinical staff. The time to initial ECG was longer following the introduction of the LACPP and was much longer than the recommended 10 min by the ESC.^7^ Dedicated ECG technicians based in ED will lead to improvements here.

The introduction of the LACPP reduced the number of patients having multiple troponins. This was in part due to the inclusion the single sample rule out pathway for patients with pain >3 hours and an initial hs-TnT of <5 ng/L, the LOD of the assay.

A meta-analysis of 11 cohorts with a pooled study of population of 9241 patients, by Pickering *et al*^5^ revealed that an initial hs-TnT<5 ng/L (LOD) and a non-ischaemic ECG gave a pooled sensitivity of 98.7% (95% CI 96.6% to 99.5%) and negative predictive value of 99.3% (95% CI 97.3% to 99.8%). These were favourable results but below the consensus goal of 99.5% for negative predictive value. In this study, of the 14 false negatives in the population, 7 were in patients whose chest pain onset was <3 hours to troponin draw. This highlights the challenges of early presenters with rapid rule-out strategies. Careful consideration is needed to discharge based on a single sample, particularly if the onset is <2 hours before blood draw. Therefore, the LACPP advocates a repeat troponin sample at 3 hours even if the initial is below the LOD.

Reduced need for repeat sampling has economic benefits. Not only does it reduce ED LOS, but it reduces work load in the biochemistry laboratory. It avoids unnecessary testing, which may in turn reduce burden and have a positive effect on lab result turnaround time.

### Limitations

The data collected are not randomised. There were differences in patient complexity and demographics between the two cohorts. The 2011 cohort was higher risk, and therefore, comparing discharge rates will be confounded by these differences. Clinical practice changes over time, including discharge decision-making. Given the difference in patient population risk factors, the ADP may not be the only factor influencing discharge. Discharge from ED did almost double, however.

We do not have data regarding rates of angiography and intervention in either cohort. Therefore, we cannot draw conclusions regarding the appropriateness of invasive intervention in those ruled-in based on troponin results.

The 2011 data for this QIP were collected retrospectively. Therefore, not all the recorded information was available and documented for each patient and not all patients were included in analysis for every variable.

The study analysed data up to 30 days post admission. Therefore, we are unable to comment on the safety of the LACPP beyond this period.

### Strengths

Data from 3003 patients across two cohorts constitutes a large sample of real-world practice. It is an accurate representation of a busy ED. Patient inclusion was also consecutive with a 100% follow-up rate, nationally in the 2011 cohort and regionally in 2018 cohort. The size of this sample and the unselected, consecutive nature of data collection indicates generalisability of these findings. Patients diagnosed with MI were adjudicated by two physicians, to ensure accuracy of diagnosis. This level of quality control is something rarely seen in QIPs.

## Conclusion

In patients presenting to ED with symptoms suggestive of ACS, our data suggest that, early discharge is significantly enhanced with implementation of the ADP without any significant harm. However, maximal potential of the ADP can only be achieved by continued improvements in healthcare provision, staff education and adequate resources.

## Data Availability

Data are available on reasonable request. Data used for statistical analysis is available for review on request.
