# Supplementary material for: Implementation of the European Society of Cardiology 0/3-hour accelerated diagnostic protocol, using high sensitive troponin T: a clinical practice evaluation of safety and effectiveness involving 3003 patients with suspected acute coronary syndrome
Source: Open Heart. 2023 Dec 26;10(2):e002366. doi: 10.1136/openhrt-2023-002366 (PMC10753736; doi:10.1136/openhrt-2023-002366)
Supplement: Supplementary data [file openhrt-2023-002366supp001.pdf]

Table s1

ICD-10 codes for chest pain, coronary artery disease and revascularisation used to extract follow-up data.

CLINICAL_CODE	CODE_SET_ID	VERSION	SHORT_DESC	LONG_DESC	OPCS_VERSION
K40	OPCS	4	SAPHENOUS VEIN GRAFT REPLACEME	SAPHENOUS VEIN GRAFT REPLACEMENT OF CORONARY ARTERY	4.2
K401	OPCS	4	Saphenous vein graft replaceme	Saphenous vein graft replacement of one coronary artery	4.2
K4010	OPCS	4	Coronary Artery Bypass Grafts	SAPHENOUS VEIN GRAFT REPLACEMENT OF ONE CORONARY ARTERY	4.2
K4010B10	OPCS	4	CORONARY ARTERY BYPASS GRAFTS	SAPHENOUS VEIN GRAFT REPLACEMENT OF ONE CORONARY ARTERY	4.2
K4010B11	OPCS	4	Coronary Artery Bypass Grafts	SAPHENOUS VEIN GRAFT REPLACEMENT OF ONE CORONARY ARTERY	4.2
K4010B14	OPCS	4	CORONARY BYPASS GRAFTS - ENDO	SAPHENOUS VEIN GRAFT REPLACEMENT OF ONE CORONARY ARTERY	4.2
K4010B16	OPCS	4	REPAIR ASC. AORTA & COR. BYPAS	SAPHENOUS VEIN GRAFT REPLACEMENT OF ONE CORONARY ARTERY	4.2
K4010B17	OPCS	4	REPAIR AORTIC ANEURYSM & BYPAS	SAPHENOUS VEIN GRAFT REPLACEMENT OF ONE CORONARY ARTERY	4.2
K4010B18	OPCS	4	CABG & OTHER MAJOR CARDIAC (EG	SAPHENOUS VEIN GRAFT REPLACEMENT OF ONE CORONARY ARTERY	4.2
K4010B1A	OPCS	4	TMR & CABG	SAPHENOUS VEIN GRAFT REPLACEMENT OF ONE CORONARY ARTERY	4.2
K4010B1B	OPCS	4	CABG & CAROTID ENDARTERECTOMY	SAPHENOUS VEIN GRAFT REPLACEMENT OF ONE CORONARY ARTERY	4.2
K4010B1C	OPCS	4	CABG & ABDOMINAL AORTIC ANEURY	SAPHENOUS VEIN GRAFT REPLACEMENT OF ONE CORONARY ARTERY	4.2
K4010B21	OPCS	4	REPLACEMENT/REPAIR OF VALVE(S)	SAPHENOUS VEIN GRAFT REPLACEMENT OF ONE CORONARY ARTERY	4.2
K4010B22	OPCS	4	Coronary Artery Bypass Grafts	SAPHENOUS VEIN GRAFT REPLACEMENT OF ONE CORONARY ARTERY	4.2
K4010B23	OPCS	4	COMPOSITE REPL AORTA,VALVE + C	SAPHENOUS VEIN GRAFT REPLACEMENT OF ONE CORONARY ARTERY	4.2
K402	OPCS	4	Saphenous vein graft replaceme	Saphenous vein graft replacement of two coronary arteries	4.2
K4020	OPCS	4	Coronary Artery Bypass Grafts	SAPHENOUS VEIN GRAFT REPLACEMENT OF TWO CORONARY ARTERIES	4.2
K4020B10	OPCS	4	CORONARY ARTERY BYPASS GRAFTS	SAPHENOUS VEIN GRAFT REPLACEMENT OF TWO CORONARY ARTERIES	4.2
K4020B11	OPCS	4	Coronary Artery Bypass Grafts	SAPHENOUS VEIN GRAFT REPLACEMENT OF TWO CORONARY ARTERIES	4.2
K4020B14	OPCS	4	CORONARY BYPASS GRAFTS - ENDO	SAPHENOUS VEIN GRAFT REPLACEMENT OF TWO CORONARY ARTERIES	4.2
K4020B16	OPCS	4	REPAIR ASC. AORTA & COR. BYPAS	SAPHENOUS VEIN GRAFT REPLACEMENT OF TWO CORONARY ARTERIES	4.2
K4020B17	OPCS	4	REPAIR AORTIC ANEURYSM & BYPAS	SAPHENOUS VEIN GRAFT REPLACEMENT OF TWO CORONARY ARTERIES	4.2
K4020B18	OPCS	4	CABG & OTHER MAJOR CARDIAC (EG	SAPHENOUS VEIN GRAFT REPLACEMENT OF TWO CORONARY ARTERIES	4.2
K4020B1A	OPCS	4	TMR & CABG	SAPHENOUS VEIN GRAFT REPLACEMENT OF TWO CORONARY ARTERIES	4.2
K4020B1B	OPCS	4	CABG & CAROTID ENDARTERECTOMY	SAPHENOUS VEIN GRAFT REPLACEMENT OF TWO CORONARY ARTERIES	4.2
K4020B1C	OPCS	4	CABG & ABDOMINAL AORTIC ANEURY	SAPHENOUS VEIN GRAFT REPLACEMENT OF TWO CORONARY ARTERIES	4.2
K4020B21	OPCS	4	REPLACEMENT/REPAIR OF VALVE(S)	SAPHENOUS VEIN GRAFT REPLACEMENT OF TWO CORONARY ARTERIES	4.2
K4020B22	OPCS	4	CABG + AVR + Carotid Endartere	SAPHENOUS VEIN GRAFT REPLACEMENT OF TWO CORONARY ARTERIES	4.2
K4020B23	OPCS	4	COMPOSITE REPL AORTA,VALVE + C	SAPHENOUS VEIN GRAFT REPLACEMENT OF TWO CORONARY ARTERIES	4.2
K4020B32	OPCS	4	Coronary Artery Bypass Grafts	SAPHENOUS VEIN GRAFT REPLACEMENT OF TWO CORONARY ARTERIES	4.2
K403	OPCS	4	Saphenous vein graft replaceme	Saphenous vein graft replacement of three coronary arteries	4.2
K4030	OPCS	4	Coronary Artery Bypass Grafts	SAPHENOUS VEIN GRAFT REPLACEMENT OF THREE CORONARY ARTERIES	4.2
K4030B10	OPCS	4	CORONARY ARTERY BYPASS GRAFTS	SAPHENOUS VEIN GRAFT REPLACEMENT OF THREE CORONARY ARTERIES	4.2
K4030B11	OPCS	4	Coronary Artery Bypass Grafts	SAPHENOUS VEIN GRAFT REPLACEMENT OF THREE CORONARY ARTERIES	4.2
K4030B14	OPCS	4	CORONARY BYPASS GRAFTS - ENDO	SAPHENOUS VEIN GRAFT REPLACEMENT OF THREE CORONARY ARTERIES	4.2
K4030B16	OPCS	4	REPAIR ASC. AORTA & COR. BYPAS	SAPHENOUS VEIN GRAFT REPLACEMENT OF THREE CORONARY ARTERIES	4.2

K4030B18	OPCS	4	CABG & OTHER MAJOR CARDIAC (EG	SAPHENOUS VEIN GRAFT REPLACEMENT OF THREE CORONARY ARTERIES	4.2
K4030B1A	OPCS	4	TMR & CABG	SAPHENOUS VEIN GRAFT REPLACEMENT OF THREE CORONARY ARTERIES	4.2
K4030B1B	OPCS	4	CABG & CAROTID ENDARTERECTOMY	SAPHENOUS VEIN GRAFT REPLACEMENT OF THREE CORONARY ARTERIES	4.2
K4030B1C	OPCS	4	CABG & ABDOMINAL AORTIC ANEURY	SAPHENOUS VEIN GRAFT REPLACEMENT OF THREE CORONARY ARTERIES	4.2
K4030B21	OPCS	4	REPLACEMENT/REPAIR OF VALVE(S)	SAPHENOUS VEIN GRAFT REPLACEMENT OF THREE CORONARY ARTERIES	4.2
K4030B22	OPCS	4	CABG + AVR + Carotid Endartere	SAPHENOUS VEIN GRAFT REPLACEMENT OF THREE CORONARY ARTERIES	4.2
K4030B23	OPCS	4	COMPOSITE REPL AORTA,VALVE + C	SAPHENOUS VEIN GRAFT REPLACEMENT OF THREE CORONARY ARTERIES	4.2
K4030B42	OPCS	4	Coronary Artery Bypass Grafts	SAPHENOUS VEIN GRAFT REPLACEMENT OF THREE CORONARY ARTERIES	4.2
K404	OPCS	4	Saphenous vein graft replaceme	Saphenous vein graft replacement of four or more coronary ar	4.2
K4040	OPCS	4	Coronary Artery Bypass Grafts	SAPHENOUS VEIN GRAFT REPLACEMENT OF FOUR OR MORE CORONARY AR	4.2
K4040B10	OPCS	4	CORONARY ARTERY BYPASS GRAFTS	SAPHENOUS VEIN GRAFT REPLACE FOUR OR MORE CORONARY ARTERIES	4.2
K4040B11	OPCS	4	CORONARY ARTERY BYPASS GRAFTS	SAPHENOUS VEIN GRAFT REPLACE FOUR OR MORE CORONARY ARTERIES	4.2
K4040B14	OPCS	4	CORONARY BYPASS GRAFTS - ENDO	SAPHENOUS VEIN GRAFT REPLACE FOUR OR MORE CORONARY ARTERIES	4.2
K4040B16	OPCS	4	REPAIR ASC. AORTA & COR. BYPAS	SAPHENOUS VEIN GRAFT REPLACE FOUR OR MORE CORONARY ARTERIES	4.2
K4040B18	OPCS	4	CABG & OTHER MAJOR CARDIAC (EG	SAPHENOUS VEIN GRAFT REPLACE FOUR OR MORE CORONARY ARTERIES	4.2
K4040B1A	OPCS	4	TMR & CABG	SAPHENOUS VEIN GRAFT REPLACE FOUR OR MORE CORONARY ARTERIES	4.2
K4040B1B	OPCS	4	CABG & CAROTID ENDARTERECTOMY	SAPHENOUS VEIN GRAFT REPLACE FOUR OR MORE CORONARY ARTERIES	4.2
K4040B21	OPCS	4	REPLACEMENT/REPAIR OF VALVE(S)	SAPHENOUS VEIN GRAFT REPLACE FOUR OR MORE CORONARY ARTERIES	4.2
K4040B22	OPCS	4	CABG + AVR + CAROTID ENDARTERE	SAPHENOUS VEIN GRAFT REPLACE FOUR OR MORE CORONARY ARTERIES	4.2
K4040B23	OPCS	4	COMPOSITE REPL AORTA,VALVE + C	SAPHENOUS VEIN GRAFT REPLACE FOUR OR MORE CORONARY ARTERIES	4.2
K4040B52	OPCS	4	Coronary Artery Bypass Grafts	SAPHENOUS VEIN GRAFT REPLACEMENT OF FOUR OR MORE CORONARY AR	4.2
K408	OPCS	4	Other specified saphenous vein	Other specified saphenous vein graft replacement of coronary	4.2
K409	OPCS	4	Unspecified saphenous vein gra	Unspecified saphenous vein graft replacement of coronary art	4.2
K41	OPCS	4	OTHER AUTOGRAFT REPLACEMENT OF	OTHER AUTOGRAFT REPLACEMENT OF CORONARY ARTERY	4.2
K411	OPCS	4	Autograft replacement of one c	Autograft replacement of one coronary artery NEC	4.2
K4110	OPCS	4	CABG + AVR + Carotid Endartere	AUTOGRAFT REPLACEMENT OF ONE CORONARY ARTERY NEC	4.2
K4110B10	OPCS	4	CORONARY ARTERY BYPASS GRAFTS	AUTOGRAFT REPLACEMENT OF ONE CORONARY ARTERY NEC	4.2
K4110B11	OPCS	4	Coronary Artery Bypass Grafts	AUTOGRAFT REPLACEMENT OF ONE CORONARY ARTERY NEC	4.2
K4110B1A	OPCS	4	TMR & CABG	AUTOGRAFT REPLACEMENT OF ONE CORONARY ARTERY NEC	4.2
K4110B21	OPCS	4	AUTO REPLACE ONE CON ART	AUTOGRAFT REPLACEMENT OF ONE CORONARY ARTERY NEC	4.2
K4110B22	OPCS	4	CABG + AVR + Carotid Endartere	AUTOGRAFT REPLACEMENT OF ONE CORONARY ARTERY NEC	4.2
K412	OPCS	4	Autograft replacement of two c	Autograft replacement of two coronary arteries NEC	4.2
K4120	OPCS	4	CABG + AVR + Carotid Endartere	AUTOGRAFT REPLACEMENT OF TWO CORONARY ARTERIES NEC	4.2
K4120B10	OPCS	4	CORONARY ARTERY BYPASS GRAFTS	AUTOGRAFT REPLACEMENT OF TWO CORONARY ARTERIES NEC	4.2
K4120B11	OPCS	4	Coronary Artery Bypass Grafts	AUTOGRAFT REPLACEMENT OF TWO CORONARY ARTERIES NEC	4.2
K4120B1A	OPCS	4	TMR & CABG	AUTOGRAFT REPLACEMENT OF TWO CORONARY ARTERIES NEC	4.2
K4120B21	OPCS	4	AUTO REPLACE TWO CON ART	AUTOGRAFT REPLACEMENT OF TWO CORONARY ARTERIES NEC	4.2
K4120B22	OPCS	4	CABG + AVR + Carotid Endartere	AUTOGRAFT REPLACEMENT OF TWO CORONARY ARTERIES NEC	4.2
K413	OPCS	4	Autograft replacement of three	Autograft replacement of three coronary arteries NEC	4.2
K4130	OPCS	4	CABG + AVR + Carotid Endartere	AUTOGRAFT REPLACEMENT OF THREE CORONARY ARTERIES NEC	4.2
K4130B10	OPCS	4	CORONARY ARTERY BYPASS GRAFTS	AUTOGRAFT REPLACEMENT OF THREE CORONARY ARTERIES NEC	4.2

K4130B11	OPCS	4	Coronary Artery Bypass Grafts	AUTOGRAFT REPLACEMENT OF THREE CORONARY ARTERIES NEC	4.2
K4130B1A	OPCS	4	TMR & CABG	AUTOGRAFT REPLACEMENT OF THREE CORONARY ARTERIES NEC	4.2
K4130B21	OPCS	4	AUTO REPLACE THREE CON ART	AUTOGRAFT REPLACEMENT OF THREE CORONARY ARTERIES NEC	4.2
K4130B22	OPCS	4	CABG + AVR + Carotid Endartere	AUTOGRAFT REPLACEMENT OF THREE CORONARY ARTERIES NEC	4.2
K414	OPCS	4	Autograft replacement of four	Autograft replacement of four or more coronary arteries NEC	4.2
K4140	OPCS	4	CABG + AVR + Carotid Endartere	AUTOGRAFT REPLACEMENT OF FOUR OR MORE CORONARY ARTERIES NEC	4.2
K4140B10	OPCS	4	CORONARY ARTERY BYPASS GRAFTS	AUTOGRAFT REPLACEMENT OF FOUR OR MORE CORONARY ARTERIES NEC	4.2
K4140B11	OPCS	4	Coronary Artery Bypass Grafts	AUTOGRAFT REPLACEMENT OF FOUR OR MORE CORONARY ARTERIES NEC	4.2
K4140B1A	OPCS	4	TMR & CABG	AUTOGRAFT REPLACEMENT OF FOUR OR MORE CORONARY ARTERIES NEC	4.2
K4140B21	OPCS	4	AUTO REPLACE FOUR > CON ART	AUTOGRAFT REPLACEMENT OF FOUR OR MORE CORONARY ARTERIES NEC	4.2
K4140B22	OPCS	4	CABG + AVR + Carotid Endartere	AUTOGRAFT REPLACEMENT OF FOUR OR MORE CORONARY ARTERIES NEC	4.2
K418	OPCS	4	Other specified other autograf	Other specified other autograft replacement of coronary arte	4.2
K4180	OPCS	4	TMR & CABG	OTHER SPECIFIED	4.2
K4180B10	OPCS	4	CORONARY ARTERY BYPASS GRAFTS	OTHER AUTOGRAFT REPLACEMENT OF CORONARY ARTERY OS	4.2
K4180B11	OPCS	4	CORONARY ARTERY BYPASS GRAFTS	OTHER AUTOGRAFT REPLACEMENT OF CORONARY ARTERY OS	4.2
K4180B1A	OPCS	4	TMR & CABG	OTHER AUTOGRAFT REPLACEMENT OF CORONARY ARTERY OS	4.2
K419	OPCS	4	Unspecified other autograft re	Unspecified other autograft replacement of coronary artery	4.2
K4190	OPCS	4	TMR & CABG	UNSPECIFIED	4.2
K4190B10	OPCS	4	CORONARY ARTERY BYPASS GRAFTS	OTHER AUTOGRAFT REPLACEMENT OF CORONARY ARTERY UNSPECIFIED	4.2
K4190B11	OPCS	4	CORONARY ARTERY BYPASS GRAFTS	OTHER AUTOGRAFT REPLACEMENT OF CORONARY ARTERY UNSPECIFIED	4.2
K4190B1A	OPCS	4	TMR & CABG	OTHER AUTOGRAFT REPLACEMENT OF CORONARY ARTERY UNSPECIFIED	4.2
K42	OPCS	4	ALLOGRAFT REPLACEMENT OF CORON	ALLOGRAFT REPLACEMENT OF CORONARY ARTERY	4.2
K421	OPCS	4	Allograft replacement of one c	Allograft replacement of one coronary artery	4.2
K422	OPCS	4	Allograft replacement of two c	Allograft replacement of two coronary arteries	4.2
K423	OPCS	4	Allograft replacement of three	Allograft replacement of three coronary arteries	4.2
K424	OPCS	4	Allograft replacement of four	Allograft replacement of four or more coronary arteries	4.2
K428	OPCS	4	Other specified allograft repl	Other specified allograft replacement of coronary artery	4.2
K429	OPCS	4	Unspecified allograft replacem	Unspecified allograft replacement of coronary artery	4.2
K43	OPCS	4	PROSTHETIC REPLACEMENT OF CORO	PROSTHETIC REPLACEMENT OF CORONARY ARTERY	4.2
K431	OPCS	4	Prosthetic replacement of one	Prosthetic replacement of one coronary artery	4.2
K432	OPCS	4	Prosthetic replacement of two	Prosthetic replacement of two coronary arteries	4.2
K433	OPCS	4	Prosthetic replacement of thre	Prosthetic replacement of three coronary arteries	4.2
K434	OPCS	4	Prosthetic replacement of four	Prosthetic replacement of four or more coronary arteries	4.2
K438	OPCS	4	Other specified prosthetic rep	Other specified prosthetic replacement of coronary artery	4.2
K439	OPCS	4	Unspecified prosthetic replace	Unspecified prosthetic replacement of coronary artery	4.2
K44	OPCS	4	OTHER REPLACEMENT OF CORONARY	OTHER REPLACEMENT OF CORONARY ARTERY	4.2
K441	OPCS	4	Replacement of coronary arteri	Replacement of coronary arteries using multiple methods	4.2
K442	OPCS	4	Revision of replacement of cor	Revision of replacement of coronary artery	4.2
K4420	OPCS	4	POST OP ATTENTION TO GRAFTS -	REVISION OF REPLACEMENT OF CORONARY ARTERY	4.2
K4420B15	OPCS	4	POST OP ATTENTION TO GRAFTS -	REVISION OF REPLACEMENT OF CORONARY ARTERY	4.2
K448	OPCS	4	Other specified other replacem	Other specified other replacement of coronary artery	4.2
K449	OPCS	4	Unspecified other replacement	Unspecified other replacement of coronary artery	4.2

K451	OPCS	4	Double anastomosis of mammary	Double anastomosis of mammary arteries to coronary arteries	4.2
K4510B10	OPCS	4	DOUBLE ANASTOMOSIS OF MAMMARY	DOUBLE ANASTOMOSIS OF MAMMARY ARTERIES TO CORONARY ARTERIES	4.2
K4510B11	OPCS	4	DOUBLE ANASTOMOSIS OF MAMMARY	DOUBLE ANASTOMOSIS OF MAMMARY ARTERIES TO CORONARY ARTERIES	4.2
K4510B21	OPCS	4	CABG WITH VALVE REPAIR	DOUBLE ANASTOMOSIS OF MAMMARY ARTERIES TO CORONARY ARTERIES	4.2
K452	OPCS	4	Double anastomosis of thoracic	Double anastomosis of thoracic arteries to coronary arteries	4.2
K453	OPCS	4	Anastomosis of mammary artery	Anastomosis of mammary artery to left anterior descending co	4.2
K4530	OPCS	4	Coronary Artery Bypass Grafts	ANASTOMOSIS OF MAMMARY ARTERY TO LEFT ANTERIOR DESCENDING CO	4.2
K4530B10	OPCS	4	CORONARY ARTERY BYPASS GRAFTS	ANAST MAMMARY ARTERY TO LT ANTERIOR DESCENDING CORON ARTERY	4.2
K4530B11	OPCS	4	CORONARY ARTERY BYPASS GRAFTS	ANAST MAMMARY ARTERY TO LT ANTERIOR DESCENDING CORON ARTERY	4.2
K4530B13	OPCS	4	MIDCAB-MIN INVASIVE BYPASS GRA	ANAST MAMMARY ARTERY TO LT ANTERIOR DESCENDING CORON ARTERY	4.2
K4530B14	OPCS	4	CORONARY BYPASS GRAFTS - ENDO	ANAST MAMMARY ARTERY TO LT ANTERIOR DESCENDING CORON ARTERY	4.2
K4530B16	OPCS	4	REPAIR ASC. AORTA & COR. BYPAS	ANAST MAMMARY ARTERY TO LT ANTERIOR DESCENDING CORON ARTERY	4.2
K4530B18	OPCS	4	CABG & OTHER MAJOR CARDIAC (EG	ANAST MAMMARY ARTERY TO LT ANTERIOR DESCENDING CORON ARTERY	4.2
K4530B1A	OPCS	4	TMR & CABG	ANAST MAMMARY ARTERY TO LT ANTERIOR DESCENDING CORON ARTERY	4.2
K4530B1B	OPCS	4	CABG & CAROTID ENDARTERECTOMY	ANAST MAMMARY ARTERY TO LT ANTERIOR DESCENDING CORON ARTERY	4.2
K4530B21	OPCS	4	REPLACEMENT/REPAIR OF VALVE(S)	ANAST MAMMARY ARTERY TO LT ANTERIOR DESCENDING CORON ARTERY	4.2
K4530B22	OPCS	4	CABG + AVR + CAROTID ENDARTERE	ANAST MAMMARY ARTERY TO LT ANTERIOR DESCENDING CORON ARTERY	4.2
K4530B23	OPCS	4	COMPOSITE REPL AORTA,VALVE + C	ANAST MAMMARY ARTERY TO LT ANTERIOR DESCENDING CORON ARTERY	4.2
K4530B62	OPCS	4	Coronary Artery Bypass Grafts	ANASTOMOSIS OF MAMMARY ARTERY TO LEFT ANTERIOR DESCENDING CO	4.2
K454	OPCS	4	Anastomosis of mammary artery	Anastomosis of mammary artery to coronary artery NEC	4.2
K4540	OPCS	4	Coronary Artery Bypass Grafts	ANASTOMOSIS OF MAMMARY ARTERY TO CORONARY ARTERY NEC	4.2
K4540B10	OPCS	4	CORONARY ARTERY BYPASS GRAFTS	ANASTOMOSIS OF MAMMARY ARTERY TO CORONARY ARTERY NEC	4.2
K4540B11	OPCS	4	Coronary Artery Bypass Grafts	ANASTOMOSIS OF MAMMARY ARTERY TO CORONARY ARTERY NEC	4.2
K4540B12	OPCS	4	Coronary Artery Bypass Grafts	ANASTOMOSIS OF MAMMARY ARTERY TO CORONARY ARTERY NEC	4.2
K4540B14	OPCS	4	CORONARY BYPASS GRAFTS - ENDO	ANASTOMOSIS OF MAMMARY ARTERY TO CORONARY ARTERY NEC	4.2
K4540B16	OPCS	4	REPAIR ASC. AORTA & COR. BYPAS	ANASTOMOSIS OF MAMMARY ARTERY TO CORONARY ARTERY NEC	4.2
K4540B18	OPCS	4	CABG & OTHER MAJOR CARDIAC (EG	ANASTOMOSIS OF MAMMARY ARTERY TO CORONARY ARTERY NEC	4.2
K4540B1A	OPCS	4	TMR & CABG	ANASTOMOSIS OF MAMMARY ARTERY TO CORONARY ARTERY NEC	4.2
K4540B1B	OPCS	4	CABG & CAROTID ENDARTERECTOMY	ANASTOMOSIS OF MAMMARY ARTERY TO CORONARY ARTERY NEC	4.2
K4540B21	OPCS	4	REPLACE/REPAIR VALVE(S) & CABG	ANASTOMOSIS OF MAMMARY ARTERY TO CORONARY ARTERY NEC	4.2
K4540B22	OPCS	4	CABG + AVR + Carotid Endartere	ANASTOMOSIS OF MAMMARY ARTERY TO CORONARY ARTERY NEC	4.2
K4540B23	OPCS	4	COMPOSITE REPL AORTA,VALVE + C	ANASTOMOSIS OF MAMMARY ARTERY TO CORONARY ARTERY NEC	4.2
K455	OPCS	4	Anastomosis of thoracic artery	Anastomosis of thoracic artery to coronary artery NEC	4.2
K456	OPCS	4	Revision of connection of thor	Revision of connection of thoracic artery to coronary artery	4.2
K458	OPCS	4	Other specified connection of	Other specified connection of thoracic artery to coronary ar	4.2
K459	OPCS	4	Unspecified connection of thor	Unspecified connection of thoracic artery to coronary artery	4.2
K46	OPCS	4	OTHER BYPASS OF CORONARY ARTER	OTHER BYPASS OF CORONARY ARTERY	4.2
K463	OPCS	4	Implantation of mammary artery	Implantation of mammary artery into heart NEC	4.2
K468	OPCS	4	Other specified other bypass o	Other specified other bypass of coronary artery	4.2
K469	OPCS	4	Unspecified other bypass of co	Unspecified other bypass of coronary artery	4.2
K49	OPCS	4	TRANSLUMINAL BALLOON ANGIOPLAS	TRANSLUMINAL BALLOON ANGIOPLASTY OF CORONARY ARTERY	4.2

K491	OPCS	4	Percutaneous transluminal ball	Percutaneous transluminal balloon angioplasty of one coronar	4.2
K4910	OPCS	4	LASER ANGIOPLASTY - ONE BALLOO	PERCUTANEOUS TRANSLUMINAL BALLOON ANGIOPLASTY OF ONE CORONAR	4.2
K4910D11	OPCS	4	ABANDONED PTCA	PERCUT TRANSLUMINAL BALLOON ANGIOPLASTY ONE CORONARY ARTERY	4.2
K4910D12	OPCS	4	SIMPLE PTCA - ONE OR TWO BALLO	PERCUT TRANSLUMINAL BALLOON ANGIOPLASTY ONE CORONARY ARTERY	4.2
K4910D21	OPCS	4	SIMPLE PTCA-MORE THAN 2 BALLOO	PERCUT TRANSLUMINAL BALLOON ANGIOPLASTY ONE CORONARY ARTERY	4.2
K4910D34	OPCS	4	PTCA - ONE STENT & ONE BALLOON	PERCUT TRANSLUMINAL BALLOON ANGIOPLASTY ONE CORONARY ARTERY	4.2
K4910D35	OPCS	4	PTCA - 1 STENT, NO BALLOON	PERCUT TRANSLUMINAL BALLOON ANGIOPLASTY ONE CORONARY ARTERY	4.2
K4910D37	OPCS	4	LASER ANGIOPLASTY - ONE BALLOO	PERCUT TRANSLUMINAL BALLOON ANGIOPLASTY ONE CORONARY ARTERY	4.2
K4910D41	OPCS	4	PTCA - 2 OR MORE STENTS	PERCUT TRANSLUMINAL BALLOON ANGIOPLASTY ONE CORONARY ARTERY	4.2
K4910D42	OPCS	4	PTCA - 2 OR MORE STENTS, NO BA	PERCUT TRANSLUMINAL BALLOON ANGIOPLASTY ONE CORONARY ARTERY	4.2
K4910D44	OPCS	4	PTCA WITH STENT & ROTABLATOR	PERCUT TRANSLUMINAL BALLOON ANGIOPLASTY ONE CORONARY ARTERY	4.2
K4910D45	OPCS	4	PTCA WITH STENT - 2+ BALLOONS	PERCUT TRANSLUMINAL BALLOON ANGIOPLASTY ONE CORONARY ARTERY	4.2
K4910D49	OPCS	4	PTCA WITH 2+STENTS (2+ BALLOON	PERCUT TRANSLUMINAL BALLOON ANGIOPLASTY ONE CORONARY ARTERY	4.2
K4910D4B	OPCS	4	LASER ANGIOPLASTY, MORE THAN O	PERCUT TRANSLUMINAL BALLOON ANGIOPLASTY ONE CORONARY ARTERY	4.2
K4910D4C	OPCS	4	LASER ANGIOPLASTY WITH STENT(S	PERCUT TRANSLUMINAL BALLOON ANGIOPLASTY ONE CORONARY ARTERY	4.2
K492	OPCS	4	Percutaneous transluminal ball	Percutaneous transluminal balloon angioplasty of multiple co	4.2
K4920	OPCS	4	PTCA with Stent & Rotablator	PERCUTANEOUS TRANSLUMINAL BALLOON ANGIOPLASTY OF MULTIPLE CO	4.2
K4920D11	OPCS	4	ABANDONED PTCA	PERCUT TRANSLUM BALLOON ANGIOPLASTY MULITPLE CORON ARTERIES	4.2
K4920D12	OPCS	4	SIMPLE PTCA - ONE OR TWO BALLO	PERCUT TRANSLUM BALLOON ANGIOPLASTY MULITPLE CORON ARTERIES	4.2
K4920D21	OPCS	4	SIMPLE PTCA-MORE THAN 2 BALLOO	PERCUT TRANSLUM BALLOON ANGIOPLASTY MULITPLE CORON ARTERIES	4.2
K4920D34	OPCS	4	PTCA - ONE STENT & ONE BALLOON	PERCUT TRANSLUM BALLOON ANGIOPLASTY MULITPLE CORON ARTERIES	4.2
K4920D35	OPCS	4	PTCA - 1 STENT, NO BALLOON	PERCUT TRANSLUM BALLOON ANGIOPLASTY MULITPLE CORON ARTERIES	4.2
K4920D37	OPCS	4	LASER ANGIOPLASTY - 1 BALLOON	PERCUT TRANSLUM BALLOON ANGIOPLASTY MULITPLE CORON ARTERIES	4.2
K4920D41	OPCS	4	PTCA - 2 OR MORE STENTS	PERCUT TRANSLUM BALLOON ANGIOPLASTY MULITPLE CORON ARTERIES	4.2
K4920D42	OPCS	4	PTCA - 2 STENTS, NO BALLOON	PERCUT TRANSLUM BALLOON ANGIOPLASTY MULITPLE CORON ARTERIES	4.2
K4920D44	OPCS	4	PTCA WITH STENT & ROTABLATOR	PERCUT TRANSLUM BALLOON ANGIOPLASTY MULITPLE CORON ARTERIES	4.2
K4920D45	OPCS	4	PTCA WITH STENT - 2+ BALLOONS	PERCUT TRANSLUM BALLOON ANGIOPLASTY MULITPLE CORON ARTERIES	4.2
K4920D49	OPCS	4	PTCA WITH 2+STENTS (2+ BALLOON	PERCUT TRANSLUM BALLOON ANGIOPLASTY MULITPLE CORON ARTERIES	4.2
K4920D4B	OPCS	4	LASER ANGIOPLASTY - 1 BALLOON	PERCUT TRANSLUM BALLOON ANGIOPLASTY MULITPLE CORON ARTERIES	4.2
K4920D4C	OPCS	4	LASER ANGIOPLASTY WITH STENT(S	PERCUT TRANSLUM BALLOON ANGIOPLASTY MULITPLE CORON ARTERIES	4.2
K493	OPCS	4	Percutaneous transluminal ball	Percutaneous transluminal balloon angioplasty of bypass graf	4.2
K4930	OPCS	4	PTCA with Stent & Rotablator	PERCUTANEOUS TRANSLUMINAL BALLOON ANGIOPLASTY OF BYPASS GRAF	4.2
K4930D12	OPCS	4	SIMPLE PTCA - ONE OR TWO BALLO	PERCUT TRANSLUM BALLOON ANGIOPLASTY BYPASS GRAFT CORON ART	4.2
K4930D21	OPCS	4	SIMPLE PTCA-MORE THAN 2 BALLOO	PERCUT TRANSLUM BALLOON ANGIOPLASTY BYPASS GRAFT CORON ART	4.2
K4930D34	OPCS	4	PTCA - ONE STENT & ONE BALLOON	PERCUT TRANSLUM BALLOON ANGIOPLASTY BYPASS GRAFT CORON ART	4.2
K4930D35	OPCS	4	PTCA - 1 STENT, NO BALLOON	PERCUT TRANSLUM BALLOON ANGIOPLASTY BYPASS GRAFT CORON ART	4.2
K4930D41	OPCS	4	PTCA - 2 OR MORE STENTS	PERCUT TRANSLUM BALLOON ANGIOPLASTY BYPASS GRAFT CORON ART	4.2
K4930D44	OPCS	4	PTCA WITH STENT & ROTABLATOR	PERCUT TRANSLUM BALLOON ANGIOPLASTY BYPASS GRAFT CORON ART	4.2
K4930D45	OPCS	4	PTCA WITH STENT - 2+ BALLOONS	PERCUT TRANSLUM BALLOON ANGIOPLASTY BYPASS GRAFT CORON ART	4.2
K4930D49	OPCS	4	PTCA WITH 2+STENTS (2+ BALLOON	PERCUT TRANSLUM BALLOON ANGIOPLASTY BYPASS GRAFT CORON ART	4.2
K494	OPCS	4	Percutaneous transluminal cutt	Percutaneous transluminal cutting balloon angioplasty of cor	4.2

K498	OPCS	4	Other specified transluminal b	Other specified transluminal balloon angioplasty of coronary	4.2
K4980	OPCS	4	PTCA with Stent & Rotablator	OTHER SPECIFIED	4.2
K4980D34	OPCS	4	PTCA - ONE STENT & ONE BALLOON	TRANSLUMINAL BALLOON ANGIOPLASTY OF CORONARY ARTERY OS	4.2
K4980D41	OPCS	4	PTCA - 2 OR MORE STENTS	TRANSLUMINAL BALLOON ANGIOPLASTY OF CORONARY ARTERY OS	4.2
K4980D44	OPCS	4	PTCA WITH STENT & ROTABLATOR	TRANSLUMINAL BALLOON ANGIOPLASTY OF CORONARY ARTERY OS	4.2
K4980D45	OPCS	4	PTCA WITH STENT - 2+ BALLOONS	TRANSLUMINAL BALLOON ANGIOPLASTY OF CORONARY ARTERY OS	4.2
K4980D49	OPCS	4	PTCA WITH 2+STENTS (2+ BALLOON	TRANSLUMINAL BALLOON ANGIOPLASTY OF CORONARY ARTERY OS	4.2
K499	OPCS	4	Unspecified transluminal ballo	Unspecified transluminal balloon angioplasty of coronary art	4.2
K50	OPCS	4	OTHER THERAPEUTIC TRANSLUMINAL	OTHER THERAPEUTIC TRANSLUMINAL OPERATIONS ON CORONARY ARTERY	4.2
K501	OPCS	4	Percutaneous transluminal lase	Percutaneous transluminal laser coronary angioplasty	4.2
K5010	OPCS	4	LASER ANGIOPLASTY - NO BALLOON	PERCUTANEOUS TRANSLUMINAL LASER CORONARY ANGIOPLASTY	4.2
K5010D36	OPCS	4	LASER ANGIOPLASTY - NO BALLOON	PERCUTANEOUS TRANSLUMINAL LASER CORONARY ANGIOPLASTY	4.2
K502	OPCS	4	Percutaneous transluminal coro	Percutaneous transluminal coronary thrombolysis using strept	4.2
K503	OPCS	4	Percutaneous transluminal inje	Percutaneous transluminal injection of therapeutic substance	4.2
K504	OPCS	4	Percutaneous transluminal athe	Percutaneous transluminal atherectomy of coronary artery	4.2
K508	OPCS	4	Other specified other therapeu	Other specified other therapeutic transluminal operations on	4.2
K5080	OPCS	4	PTCA with Rota -1 balloon,1 or	PTDA	4.2
K5080D31	OPCS	4	PTCA WITH ROTA -1 BALLOON,1 OR	OTHER THERAP TRANSLUMINAL OPERATIONS ON CORONARY ARTERY OS	4.2
K5080D32	OPCS	4	CORONARY TEC - ONE BALLOON	OTHER THERAP TRANSLUMINAL OPERATIONS ON CORONARY ARTERY OS	4.2
K5080D33	OPCS	4	DCA - ONE BALLOON	OTHER THERAP TRANSLUMINAL OPERATIONS ON CORONARY ARTERY OS	4.2
K5080D43	OPCS	4	PTCA-2 OR MORE ROTABLATORS	OTHER THERAP TRANSLUMINAL OPERATIONS ON CORONARY ARTERY OS	4.2
K5080D44	OPCS	4	PTCA WITH STENT & ROTABLATOR	OTHER THERAP TRANSLUMINAL OPERATIONS ON CORONARY ARTERY OS	4.2
K5080D46	OPCS	4	PTCA WITH ROTABLATOR - 2+ BALL	OTHER THERAP TRANSLUMINAL OPERATIONS ON CORONARY ARTERY OS	4.2
K5080D47	OPCS	4	CORONARY TEC - 2+ BALLOONS	OTHER THERAP TRANSLUMINAL OPERATIONS ON CORONARY ARTERY OS	4.2
K5080D48	OPCS	4	DCA - 2+BALLOONS	OTHER THERAP TRANSLUMINAL OPERATIONS ON CORONARY ARTERY OS	4.2
K5080D4A	OPCS	4	CORONARY TEC WITH STENT(S) INC	OTHER THERAP TRANSLUMINAL OPERATIONS ON CORONARY ARTERY OS	4.2
K509	OPCS	4	Unspecified other therapeutic	Unspecified other therapeutic transluminal operations on coronary artery	4.2
K75	OPCS	4	PERCUTANEOUS TRANSLUMINAL BALL	PERCUTANEOUS TRANSLUMINAL BALLOON ANGIOPLASTY AND STENTING O	4.2
K751	OPCS	4	Percutaneous transluminal ball	Percutaneous transluminal balloon angioplasty and insertion	4.2
K752	OPCS	4	Percutaneous transluminal ball	Percutaneous transluminal balloon angioplasty and insertion	4.2
K753	OPCS	4	Percutaneous transluminal ball	Percutaneous transluminal balloon angioplasty and insertion	4.2
K754	OPCS	4	Percutaneous transluminal ball	Percutaneous transluminal balloon angioplasty and insertion	4.2
K758	OPCS	4	Other specified percutaneous t	Other specified percutaneous transluminal balloon angioplasty	4.2
K759	OPCS	4	Unspecified percutaneous trans	Unspecified percutaneous transluminal balloon angioplasty an	4.2
